# Modelling the emergence dynamics of the western corn rootworm beetle (*Diabrotica virgifera virgifera*)

**DOI:** 10.1038/s41598-022-05032-1

**Published:** 2022-02-11

**Authors:** Rodelyn Jaksons, Katharina Falkner, Elena Moltchanova

**Affiliations:** 1 The New Zealand Institute for Plant and Food Research Ltd, Private Bag 4704, Christchurch Mail Centre, Christchurch, 8140 New Zealand; 2 School of Mathematics and Statistics, University of Canterbury, Private Bag 4800, Christchurch, 8140 New Zealand; 3 Department of Economics and Social Sciences, Institute for Sustainable Economic Development, University of Natural Resources and Life Sciences, Vienna (BOKU), Feistmantelstraße 4, 1180 Vienna, Austria

**Keywords:** Environmental impact, Ecological modelling, Invasive species, Statistics

## Abstract

The western corn rootworm is an invasive species to Europe and is a major agricultural pest that causes widespread economic and yield losses to maize producers. The Gompertz curve was originally used to model human population mortality. It is a sigmoidal curve where the beginning and end of a period shows the slowest time for growth, and adequately describes observed dynamics of many phenomena. We propose the use of the Gompertz function in a Bayesian Hierarchical framework to model the emergence dynamics of the western corn rootworm beetle. The proposed model includes the use of climatic variables to assess how weather can influence the observed dynamics. We apply the model to Austrian monitoring data collected in 2004–2015.

## Introduction

Invasive species have plagued farmers since the transition from natural landscapes towards agro-ecological systems^1^. Nowadays, climate change and globalisation, i.e. global trade and transport of agricultural products, are among the most important drivers for the spread of invasive species and their introduction to new environments^1,2^. One such an invasive species is the Western Corn Rootworm (WCR; *Diabrotica virgifera virgifera*) beetle, a univoltine (one generation per growing season) leaf beetle, which is known to cause massive crop yield losses to Zea mays maize crops^3,4^. Maize is one of the most important crops in global agriculture, because of its high yield potential, the comparably low labour input, profitability and versatile use, vendibility on established markets, as well as the favourable agronomic characteristics (e.g. low requirements on crop rotations—can be planted in monocultures^5^). However, the WCR beetle is particularly problematic in intensive maize production regions, i.e. if maize is cultivated in monocultures, as it has little or no natural predators^4^.

The WCR beetle originates from North America and is believed to have been introduced to Europe at the beginning of the 1990s. It was first detected in Serbia close to Belgrade international airport in 1992, and since then the WCR infestation has continued to spread further and further year after year^6^. Within the European Union, the WCR beetle was given quarantine status in 2003^7^. After introduction of this regulation, monitoring of the WCR beetle became mandatory in infested or vulnerable locations or maize fields^8^. The monitoring was intended to assess the spread and severity of the infestation in hopes that effective pest management strategies could eradicate the population. However, eradication was not achieved. In places of long running WCR infestation the number of WCR beetles observed have increased at an alarming rate. The quarantine status of the WCR beetle was repealed in 2014^8^. In Austria, the WCR beetle was first detected in 2002^9^. WCR infestation severely disadvantages the livelihood of maize growers across Austria, whereby South-East Austria is particularly affected^10^. Most severe economic damages up to total crop yield losses were reported for about 10000 ha maize area in 2014^5^.

In order to make effective pest management decisions, it is important to understand the emergence dynamics of established WCR beetle populations. The WCR beetle is known to spend its egg stage in the soil, emerging in the late spring or early summer^3,11^. However, previous studies show that environmental factors such as temperature and precipitation influence the phenological cycle. For example, warmer temperatures increase the observed adult abundance, and WCR beetle emergence can last until the first frost. On the other hand, previous studies have reported that increased precipitation, and colder temperatures in winter as well as longer cold periods below $-10\,^{\circ }{\mathrm{C}}$ increase mortality in the overwintering eggs^12–15^. As is the case for many insects, the emergence dynamics of the WCR beetle can be described with sufficient accuracy by a parametric curve, such as, for example, the Gompertz curve.

In this study, we use the Gompertz curve to model the observed emergence dynamics of the WCR beetle. The Gompertz curve was first proposed by Benjamin Gompertz in 1825 to describe the law of human mortality^16^. The Gompertz curve is a sigmoidal curve which describes growth as being the slowest at the beginning and the end of a period. It is usually described by three parameters; an asymptote $\alpha$, a relative starting value $\beta$, and a growth rate coefficient $\gamma$. Since its introduction, the Gompertz curve has been applied in many population biology studies^17–20^.

In this study, the upper asymptote parameter $\alpha$ was considered as a proxy for the saturation level of WCR population growth. The growth rate coefficient $\gamma$ was treated as an indicator of the WCR beetles’ emergence period, with lower values of $\gamma$ indicating a more protracted period of beetle emergence.

We incorporated the above structure into a Bayesian hierarchical modelling framework and used Markov Chain Monte Carlo (MCMC) methods for parameter estimation and posterior inference. We applied the model to the WCR trapping data for Austria obtained in 2004–2015. We investigated the effect of climate covariates such as temperature and precipitation on the WCR beetle emergence dynamics. The handling of missing data and possible inclusion of spatial autocorrelation is also discussed .

The overview of the paper is as follows; In “Data” section we give an overview of the used data. “Methodology” section provides a detailed description of the methodology and the handling of missing data. The results are presented in “Results” section and discussed in “Discussion” section where we also address the need/potential for future research.

## Data

**Figure 1:**
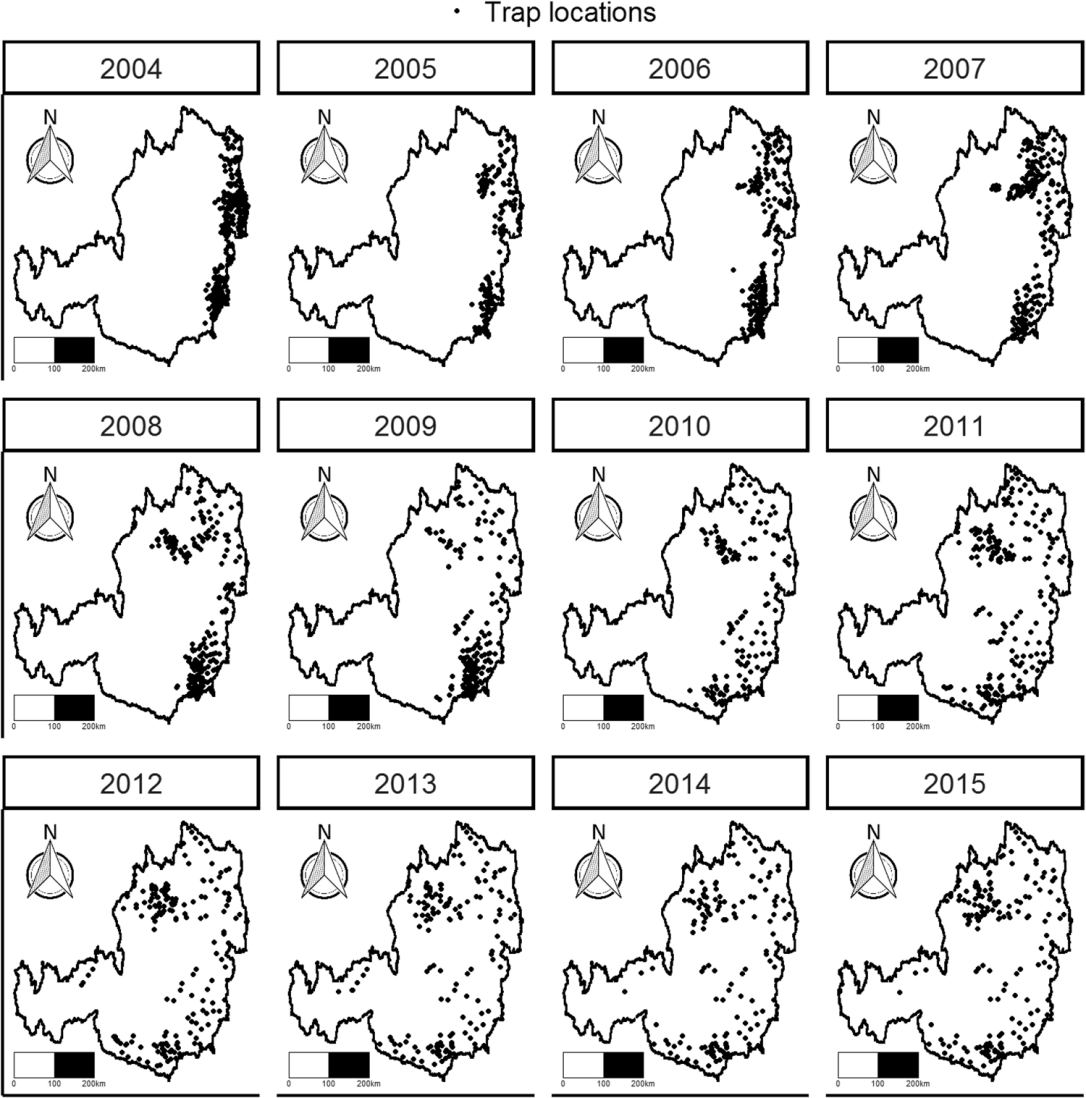
The locations of the placed traps where at least one WCR beetle was caught in 2004–2015.

The data set consists of WCR trappings recorded across maize-growing locations in Austria, as shown in Fig. 1^21^. A trap was placed at a location if the WCR beetle had been previously observed and at locations and regions that are considered vulnerable to WCR infestation^8^. The traps were laid at the beginning of the maize growing season (usually in the beginning of June) until harvest (usually the beginning of October), thus giving a monitoring period of 19 weeks. The traps operated by releasing pheromones which attracted male beetles, and yellow sticky tapes trapped the WCR beetle^22^. Each week, the number of beetles caught were counted and the traps were replaced. The WCR trapping data was collected during the monitoring periods of 2004–2015, with only observed cases recorded, i.e. traps that trapped at least one WCR beetle over the monitoring period.

Of the total of 4745 traps, monitoring WCR beetles between 2004 and 2015, a total of 2702 traps (56.9%) had at least one non-zero record and were included in the analysis. In this data set blanks or zeroes were used interchangeably, thus one could not distinguish if a record was missing or was indeed a zero count. Each of the 2702 traps consisted of 19 weeks of monitoring data, however for the first three and the last three weeks of the monitoring season, zero counts were recorded for almost all the traps. For the remaining 2702*13=35126 records, 18158, (51.7%) were recorded as either blanks or zeroes, of which 1881 suspicious blanks/zeroes were recorded in the middle of the season. Because we were modelling cumulative emergence, omitting a missing data point would mean omitting the records for the entire trap and thus loosing 2698 traps or 83.68% of the data. Instead, after a consultation with two domain experts, we set up the following scheme:

- Any blank or zero records until the first numeric entry were coded as zero, as they are likely to be zero in the beginning of the monitoring period/early season ($n=8213$, or $16\%$)
- Any blank or zero records which occurred between two non-zero entries, at least one of which was greater than or equal to 10, were re-coded as missing. ($n=78$ or $0.15\%$). Otherwise they were coded as zeroes.
- Any blank or zero records which occurred between two zero entries were coded as zeroes, as sequential zero counts are possible ($n=25$, or $0.05\%$).
- Any blank or missing record that were directly adjacent to a non-zero entry was coded as missing (i.e. at least one non-zero entry), as there is a chance that it is missing rather than a zero count ($n=3010$, or $5.86\%$).

Note, that in the Bayesian framework, missing values are treated as (nuisance) parameters. The model also includes covariates to see if emergence dynamics were associated with climatic variables, and were available for each trap location based on the nearest weather station^23^. In this work the following variables were considered; the average winter temperature (1st January–end of March), the average spring temperature (1st April–end of June), and the precipitation sum during winter (1st January–end of March) and the temporal trend for the year. Moreover, the percentage of the agricultural area per Austrian municipality used for cultivating maize crops (maize), as provided by the Austrian Gemeindedatenbank^24^ was included in the analysis.

## Methodology

Let $y_{itk}$ denote the WCR count observed for trap *i* in week *t* in year *k*, and assume it to follow a Poisson distribution with parameter $\mu _{itk}$

1 $$\begin{aligned} y_{itk} | \mu _{itk}, \sim Poisson(\mu _{itk}) \end{aligned}$$

The intensity parameter $\mu _{itk}$ represents the rate of emergence for a given time period. Instead of allowing it to depend purely on time *t*, a phenological variable of growing degree days (GDD) is used, as warmer temperatures are required for WCR development^25–28^. GDDs reflect the heat accumulation and are defined as an integral of warmth above a base temperature after a given start date:

2 $$\begin{aligned} GDD = \int (T(t)-T_{base})dt. \end{aligned}$$

The above integral can be approximated by

3 $$\begin{aligned} GDD = \max \left( \frac{T_{max} - T_{min}}{2} - T_{base}, 0 \right) . \end{aligned}$$

Here $T_{min}$ is the minimum daily temperature, $T_{max}$ is the maximum daily temperature, and $T_{base}$ is a set base temperature. In this study, the base temperature was set to $10\,^{\circ }$C, and the starting date was the beginning of April, which marks the start of the growing season in Austria.

The rate of cumulative emergence of the WCR beetle can be described by a Gompertz function. The Gompertz function is a sigmoidal function which describes growth as being slowest at the beginning and the end of a given period and is defined as

4 $$\begin{aligned} f(z_t) = \alpha \exp (-\beta \exp (-\gamma z_t)). \end{aligned}$$

where $\alpha$ is the upper asymptote, $\beta$ is a relative starting value, $\gamma$ is a growth rate coefficient which affects the slope, and $z_t$ are the cumulative growing degree days. In this study, one can consider the asymptote as proxy to the saturation level of WCR population growth. Lower values of $\beta$ suggest an earlier first emergence in the season, while lower values of $\gamma$ indicate a longer emergence period. To investigate whether there is an association between climate variables and the emergence dynamics, the Gompertz curve parameters were assumed to linearly depend on climate covariates. In this regression modelling framework, a spatially correlated residual structure can be added in either $\alpha$, $\beta$, and/or $\gamma$ if there is evidence to do so.

To reflect the nature of the emergence dynamics and to preserve the shape of the increasing Gompertz curve, the parameters of the model were restricted to positive values such that $\alpha >0$, $\beta >0$, and $\gamma >0$. The time at inflection or period of highest growth can be obtained by solving Eq. (4) for the value of *t* at which the concavity of the function changes. The time at inflection is described as:

5 $$\begin{aligned} T_z^* = \frac{\log (\beta )}{\gamma } \end{aligned}$$

The Gompertz function describes cumulative emergence. Thus to describe the marginal emergence rate, the derivative of the Gompertz function can be used instead. Consequently, as the WCR trapping data consisted of weekly counts, the rate of emergence $\mu _{itk}$ is better described by the log of the derivative of the Gompertz function

6 $$\begin{aligned} \log (\mu _{itk}) = \log (\alpha _{ik}) + \log (\gamma _{ik}) + \log (\beta _{ik}) + \gamma _i z_{itk} - \beta _{ik} \exp (-\gamma z_{itk}). \end{aligned}$$

The parameters $\alpha _{ik}$, $\beta _{ik}$ and $\gamma _{ik}$ are site and year specific such that:

7 $$\begin{aligned}&\alpha _{ik} \sim N(\mu _{\alpha _{ik}}, \tau _{\alpha }) \end{aligned}$$

8 $$\begin{aligned}&\gamma _{ik} \sim N(\mu _{\gamma _{ik}}, \tau _{\gamma }) \end{aligned}$$

9 $$\begin{aligned}&\beta _{ik} \sim N(\mu _{\beta _{ik}}, \tau _{\beta }). \end{aligned}$$

Here, $\tau _{\alpha }$, $\tau _{\beta }$, and $\tau _{\gamma }$ are the precision (inverse variance) parameters of the prior distributions for $\alpha$, $\beta$ and $\gamma$ respectively. Moreover, the means of the distributions $\mu _{\alpha _{ik}}$, $\mu _{\beta _{ik}}$, and $\mu _{\gamma _{ik}}$ can be expressed as functions of known covariates:

10 $$\begin{aligned} \mu _{\alpha _{ik}}= & {} a_{0} + {\mathbf {w}}^T X_{\alpha _{ik}}, \end{aligned}$$

11 $$\begin{aligned} \mu _{\beta _{ik}}= & {} b_{0}, \end{aligned}$$

12 $$\begin{aligned} \mu _{\gamma _{ik}}= & {} g_{0} + {\mathbf {u}}^T X_{\gamma _{ik}}. \end{aligned}$$

Here $a_{0}$ is the intercept, ${\mathbf {w}}$ is a vector of the regression coefficients, and $X_{\alpha _{ik}}$ are the location and year specific covariates. The predictors used in the regression of $\mu _{\alpha _{ik}}$ are the average winter temperature, the precipitation sum during winter, the year, the percentage of the agricultural area per Austrian municipality used for cultivating maize crops (maize), and the corresponding centred coordinates of the trap locations; *x*, *y*, and their functions $x^2$, $y^2$, and *xy*. The parameter $g_{0}$ is the intercept for the regression of $\mu _{\gamma _{ik}}$, and *u* is the corresponding regression coefficient. The predictor used for $\mu _{\gamma _ik}$ is the average yearly spring temperature.

The intercepts and regression coefficients ($\mathbf {w}$ and $\mathbf {u}$) were given non-informative normal priors *N*(0, 0.01). The precision parameters $\tau _{\alpha }$, $\tau _{\beta }$ and $\tau _{\gamma }$ were assigned prior distributions *Gamma*(0.01, 0.01).

The model was fitted using WinBUGS through the R2WinBUGS package in R^29–31^. The model was run for 20000 iterations, with a burn-in of 10000 iterations, and a thinning rate of five. Convergence was determined by visual assessments of trace plots and marginal posterior densities.

## Results

The regression coefficients of the model parameters are tabulated in Table 1. The parameter $\alpha$, which represents the proxy for the saturation level of WCR population growth was found to be positively correlated with the average winter temperature. During the study period a $1\,^{\circ }{\mathrm{C}}$ increase in the average winter temperature was associated with an average 15.38% increase. The coefficient for the year variable was also positively correlated with $\alpha$, with average increase of 11% per year.

**Table 1:** Regression coefficients of the model parameters.

Parameter	Variable	Post. mean (post. sd)	95% Cred. int
$\mu _\alpha$	Intercept	2.03 (0.257)	(1.51, 2.51)
	Winter temperature	0.143 (0.031)	(0.008, 0.021)
	Precipitation	− $3.72 \times 10^{-6}$ ($3.42 \times 10^{-6}$)	(− $1.04 \times 10^{-5}$, $2.80 \times 10^{-6}$)
	Maize	0.00014 (0.00013)	(− 0.00012, 0.000392)
	Year	0.106 (0.002)	(0.064, 0.143)
	*x*	0.069 (0.078)	(− 0.099, 0.217)
	*y*	0.175 (0.132)	(− 0.086, 0.043)
	*xy*	− 0.082 (0.105)	(− 0.305, 1.250)
	$x^2$	0.058 (0.038)	(− 0.016, 0.133)
	$y^2$	− 0.097 (0.203)	(− 0.510, 0.314)
	$\tau _\alpha$	0.149 (0.004)	(0.141, 0.158)
$\mu _\beta$	Intercept	0.209 (0.026)	(0.158, 0.257)
	$\tau _\beta$	0.672 (0.024)	(0.628, 0.722)
$\mu _\gamma$	Intercept	− 0.289 (0.014)	(− 0.325, − 0.268)
	Spring temp	0.0454 (0.0127)	(0.020, 0.069)
	$\tau _\gamma$	2.890 (0.1060)	(2.670, 3.090)

The growth rate coefficient $\gamma$ which represents the emergence rate was found to be positively correlated with the average spring temperature. During the study period a $1\,^{\circ }$C rise was associated with an average increase of 4% in the expected growth rate. Increased average spring temperature increases the growth rate coefficient which means that the asymptote is reached sooner.

**Figure 2:**
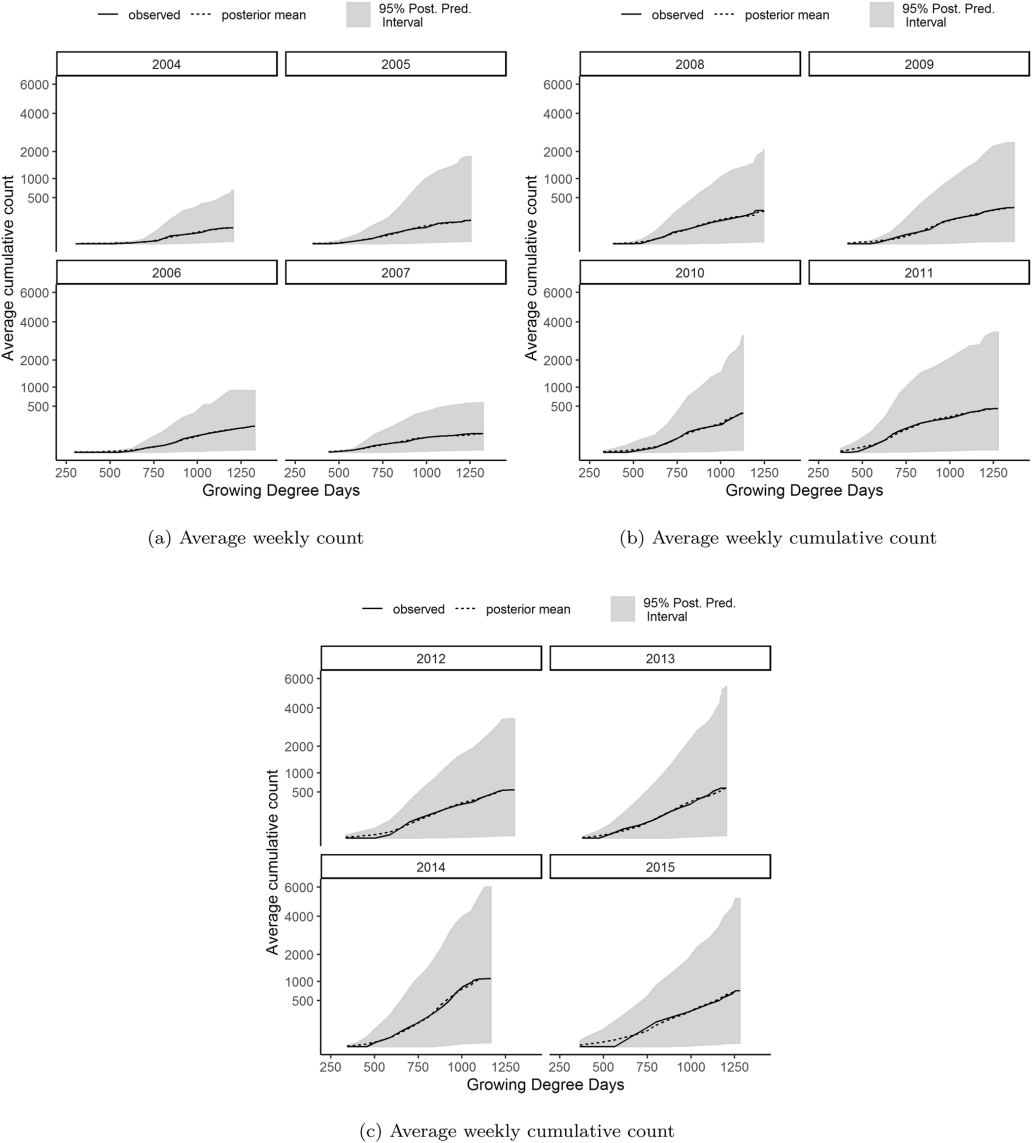
The average observed cumulative weekly WCR count and their corresponding posterior mean estimate and the 95% credible interval. The x-axis shows the monitoring week on top, and the average cumulative GDD on the bottom.

The posterior mean estimate, and the corresponding 95% credible interval for the observed mean cumulative count is depicted in Fig. 2. This figure shows that the 95% posterior predictive interval encapsulates the overall observed dynamics, and describes the data well as the posterior predictive mean estimates are similar to the average observed counts. The wide posterior predictive intervals illustrate the variability of the data itself.

## Discussion

The objective of this study was to better understand the emergence dynamics of established populations of the WCR beetle in Austria. Furthermore, it was of interest to see how climate variables such as temperature and precipitation as well as the maize production intensity (i.e. the percentage of agricultural area per Austrian municipality used for cultivating maize crops) affected the observed emergence dynamics. The results of the study showed that the saturation level of WCR population growth was most affected by temperature, and average population sizes increased each year during the study period. The rate of WCR beetle emergence was found to be positively correlated with spring temperatures, which means that peak emergence is reached earlier in the season. For growers, this is problematic as it means their maize crops are subject to higher levels of predation from the WCR beetle at early growing stages (i.e. germination of maize plants), when being particularly susceptible to damages from larval root feeding^32^. Therefore, it may be beneficial to grow maize varieties that mature later. Alternatively, maize can be planted earlier in the season, so that the rootstock is well established to better withstand damage caused by the WCR beetle, i.e. larval root feeding.

Due to climate change, the average temperatures around the world have risen and are expected to continue, and with this come new challenges to agriculture^33^. The saturation level of WCR population growth was found to be positively correlated with the average winter temperature. Therefore, the effect of warmer winters are twofold. Firstly, as a result of climate change there is likely to be an expansion of suitable habitats for WCR^34,35^. Secondly, increased temperatures and increased areas of suitable habitats are likely to result in larger WCR beetle populations and more severe infestations in upcoming growing seasons^12^.

Higher spring temperatures were also found to be associated with peak emergence occurring earlier in the growing season. This information is vital for insecticide spraying, as it is usually timed according to peak emergence so that it is most effective in reducing population size^36,37^. However, legal bans of chemical control agents in Austria (e.g. legal ban of insecticide treated seeds due to Implementing Regulation EU No (485/2013); 2013) limit the availability of pesticides and therefore farmer’s self-efficacy to control the WCR beetle^38^. Therefore, more diversified crop rotations are deemed to be the most effective WCR control measure in the long term. The adoption of these strategies is beneficial to growers as they have the best hopes of reducing yield loss. Additionally results by Feusthuber et al.^39^ indicate that crop rotations are economically more efficient for WCR control compared to insecticide applications, which is especially relevant at high crop yield loss levels from WCR infestation^39^.

Overall, the Gompertz curve have proven to be suitable for modelling the emergence dynamics of the WCR beetle in Austria. However, some limitations were faced during model development. Firstly, and most importantly, the large number of missing data (i.e. blanks and zeroes) in the data set. Because a protocol for the data recording was not available, the blanks and zeroes were used interchangeably for missing observations and actual zero counts respectively. We came up with a reasonable re-coding scheme, but other choices are possible. Moreover, the choice of sampling locations did not follow any design. A better planned study may provide more insights into the phenomena. The analysis was based on the observed WCR beetle counts for traps with at least one non-zero count during the 2004–2015 monitoring period.

The work done in this project has given valuable and practical insights on the emergence and phenological dynamics of the WCR beetle. As a result, pest management strategies can be adapted so that population control measures can be timed accordingly with peak emergence. We envision future work to include a more effective sampling study design. As the data that was used incorporated traps with at least one catch, we can incorporate the emergence model into a hurdle model which will extend the work previously done by Falkner et al.^12^.

## Data Availability

The data that support the findings of this study are available from the Austrian Agency for Health and Food Safety but restrictions apply to the availability of these data, which were used under license for the current study, and so are not publicly available. The data are not publicly available due to them containing information that could compromise research participant privacy/consent.
